# The relationship between drug-induced immunogenicity and hypersensitivity reactions and skin tests related to infliximab, etanercept and adalimumab in patients with rheumatoid arthritis and ankylosing spondylitis

**DOI:** 10.55730/1300-0144.5914

**Published:** 2024-05-16

**Authors:** Alper DOĞANCI, Şebnem ATAMAN, Ali Erhan ÖZDEMİREL, Recep Bülent SEÇKİN, Ayşe Peyman YALÇIN, Sevim BAVBEK

**Affiliations:** 1 Division of Rheumatology, Department of Physical Medicine and Rehabilitation, Faculty of Medicine, Sivas Cumhuriyet University, Sivas, Turkiye; 2 Division of Rheumatology, Department of Physical Medicine and Rehabilitation, Faculty of Medicine, Ankara University, Ankara, Turkiye; 3 Division of Rheumatology, Department of Physical Medicine and Rehabilitation, Ankara Liv Hospital, Ankara, Turkiye; 4 Department of Physical Medicine and Rehabilitation, Faculty of Medicine, Ankara University, Ankara, Turkiye; 5 Division of Clinical Immunology and Allergy, Department of Chest Diseases, Faculty of Medicine, Ankara University, Ankara, Turkiye

**Keywords:** Hypersensitivity reaction, immunogenicity, intradermal test, serum antidrug antibodies, skin prick test

## Abstract

**Background/aim:**

We aimed to investigate the relationship between serum antidrug antibodies (ADAbs), systemic hypersensitivity, or local injection site reactions to tumor necrosis factor (anti-TNF) drugs and to detect the role of skin tests in the diagnosis of hypersensitivity reactions (HSRs) against anti-TNFs.

**Materials and methods:**

Sixty-nine ankylosing spondylitis (AS) and 46 rheumatoid arthritis (RA) patients taking infliximab (IFX), adalimumab (ADA), and etanercept (ETN) were enrolled. The demographical data, erythrocyte sedimentation rate (ESR), and c-reactive protein (CRP) levels of the patients were determined, and the Bath Ankylosing Spondylitis Disease Activity Index (BASDAI) assessment for AS patients and DAS28 (disease activity score) for RA patients were assessed. Serum levels of anti-TNFs and ADAbs to these agents were measured with ELISA. These blood samples were taken 24 h before the next drug dose. The patients with anti-TNF-associated HSRs were evaluated with a skin-prick test (SPT) and intradermal test (IDT). Readings for the SPT and IDT were conducted after 15 min and 24, 48, and 72 h. Heel diameter of 3 mm or more greater than the negative control was considered positive for SPT, and if the size of the initial wheal had increased by at least 3 mm in diameter and was surrounded by erythema it was considered positive for IDT. Symptoms such as urticaria/angioedema and flushing were considered immediate-type HSRs. Findings developed at the injection site such as swelling and erythema were considered injection-site reactions (ISRs). An overall p < 0.05 was considered statistically significant.

**Results:**

A statistically significant association was found between HSRs (immediate type and ISRs) and IDT reported by patients taking biological drugs (p = 0.001). In the subgroup analysis, a statistically significant association was found between ISRs and IDT in those taking ADA and ETN (respectively p = 0.012, p = 0.013). No relationship was found between skin test positivity and the presence of IgG ADAbs to anti-TNFs or disease activity scores.

**Conclusion:**

This study demonstrates that patients who exhibit ISRs to anti-TNFs produce notably positive results to IDT without maintaining a direct relationship to serum levels of ADAbs.

## 1. Introduction

Tumor necrosis factor-alpha (TNF-α) is a proinflammatory cytokine that plays an important role in the pathogenesis of rheumatoid arthritis (RA), ankylosing spondylitis (AS), and many other inflammatory diseases. In recent years, anti-TNF drugs that antagonize the biological activities of TNF-α have been successfully used to treat rheumatic disorders.

However, the clinical response may decrease over time in some patients who have initiated treatment. The reason for the unresponsiveness to treatment is that the drug triggers the immune response (immunogenicity) after therapy starts. As a result, antibodies develop against the drug (antidrug antibodies: ADAbs). There can be two consequences of the presence of ADAbs: either the effectiveness of the drug can be decreased or hypersensitivity reactions (HSRs) can occur against the drug [1]. If ADAb occurrence did not cause these consequences, it may be a temporary condition connected with drug exposure.

Hypersensitivity reactions that occur due to TNF-α inhibitors can be systemic or local. Immediate-type HSRs are defined to occur within less than 1 h of drug administration, and they are systemic and often immunoglobulin (Ig)E mediated. They show signs such as urticaria/angioedema, flushing, generalized itching, nausea, vomiting, abdominal pain, shortness of breath, wheezing, tachycardia, and hypotension. On the other hand, injection site reactions (ISRs) are reactions that occur at the site of injection, either by irritation effect or mediated by immune mechanisms. Signs such as swelling, erythema, pruritus, and pain around the site of injection can be defined as ISRs [1].

While some of the HSRs that develop against biological agents occur mediated by ADAbs, some of them occur without the mediation of ADAbs. ADAbs can be in IgE, IgG, or IgM isotypes [2]. Among ADAb-mediated ones, the IgE-dependent ones can cause immediate reactions with the type 1 immune mechanism. On the other hand, non-IgE dependent ones can cause immediate reactions either by IgG-FcγRIII interaction with basophils, neutrophils, and macrophages or through mast cell activation via the complement system activated by immune complexes between IgG ADAbs and drug. Moreover, some of the ADAb-mediated but not IgE-dependent ones can cause delayed reactions by the immune complexes formed by IgG ADAb–drug interaction precipitating in the tissue and activating complement.

Non-ADAb-mediated reactions work in three ways. They may cause immediate/delayed reactions via the cytokine release reaction (CRR) pathway or lipids and/or aggregates activate the complement system and lead to direct mast cell activation by C3a, C5a (CARPA: complement activation related pseudoallergy) or T cell-mediated delayed reactions and ISRs. Some of the ISRs occur with irritative effects and some with immune-mediated mechanisms. Based on the shared case reports, ISRs are thought to occur with type 1-, type 3-, or type 4-mediated immune mechanisms [3].

The aim of the present study was to investigate whether there is a relationship between the development of ADAbs and drug-related HSRs during anti-TNF treatment and to examine the relationship between HSRs and serum drug levels, disease activity, and anti-TNF use in patients with RA and AS.

## 2. Materials and methods

This research was designed as a cross-sectional study. The study included 69 AS patients who met the Modified New York criteria [4] and 46 RA patients who met the 1987 American College of Rheumatology (ACR) RA criteria [5] and were followed up in the outpatient clinics of the Department of Physical Medicine and Rehabilitation and Department of Rheumatology in Ankara University Faculty of Medicine and who received anti-TNF therapy (adalimumab (ADA), etanercept (ETN), and infliximab (IFX)). The patients included in the study were confirmed to be above 18 years old. Those who took a different anti-TNF product before that was discontinued for various reasons; those who took biological agents other than ETN, ADA, and IFX during that period; those with a history of allergic disease such as anaphylaxis, chronic urticaria, asthma, and atopic dermatitis; those diagnosed with an autoimmune disease other than AS and RA; and those with active infection, malignancy, or severe organ failure were not included in the study. The demographic data (age, sex, duration of the disease, history, duration, dose of anti-TNF use, and concomitant medications) of the patients were recorded. During the follow-up in the outpatient clinic, complete blood count, erythrocyte sedimentation rate (ESR), and C-reactive protein (CRP) values were routinely checked, and serum blood was collected to determine ADAb level measurements and drug levels. For assessment of disease activity, the Bath Ankylosing Spondylitis Disease Activity Index (BASDAI) [6] for AS patients and Disease Activity Score 28 (DAS28) [7] for RA patients were measured. ISRs due to subcutaneous anti-TNFs (ADA, ETN) and immediate-type HSRs due to ADA, ETN, and IFX were investigated and recorded.

The AS patients were allowed to take NSAIDs if needed. The RA patients who could tolerate and who did not have contraindications took MTX 10 mg per week, and those who could not tolerate it and who developed contraindications took leflunomide 10 mg per day. In 5 of the 46 RA patients, disease activation occurred. For 5 days, the patients were given 40 mg of steroid in decreasing doses.

In the RA patients, RF and CCP were positive in high titers. All patients remained under treatment during the study.

Immediate HSRs are systemic reactions that occur within the first 6 h following drug administration [8], and symptoms such as urticaria/angioedema, flushing, generalized itching, nausea, vomiting, abdominal pain, shortness of breath, wheezing, palpitations, and hypotension were regarded as immediate HSRs. ISRs occur with either irritative effects or immune-mediated mechanisms [9]. Pruritus, pain, swelling, and erythema around the injection site were considered ISRs.

### 2.1. ADAbs and drug measurement

The samples obtained from the venous blood of the patients were stored at −20 °C until the analyses were performed to determine the serum levels of anti-IFX Ab, anti-ETN Ab, anti-ADA Ab, and serum drug levels. These blood samples were taken 24 h before the next drug dose in accordance with the manufacturer’s protocol. The enzyme-linked immunosorbent assay (ELISA) for the quantitative determination method was used for both the determination of serum drug levels and the measurement of ADAbs (IgG subclass 1, 2, 3) (Progenika Biopharma SA, Derio, Spain). The drug levels for IFX, ADA, and ETN were defined as follows: ≤0.035 μg/Ml (low) and ≥0.035 μg/mL (normal).

ADAb levels were defined as follows: for IFX, ≤2 AU/mL (negative) and >2 AU/mL (positive); for ADA, ≤3.5 AU/mL (negative) and >3.5 AU/mL (positive); and for ETN, ≤142 AU/mL (negative) and >142 AU/mL (positive).

### 2.2. Skin test

A skin prick test (SPT) with the full strength of the drugs was performed and was read at 15 min. A 3 mm or more wheal diameter greater than the negative control was considered positive.

In SPT-negative cases intradermal testing (IDT) was performed with dilutions of 1:1000 (0.05 mg/mL), 1:100 (0.5 mg/mL), and 1:10 (5 mg/mL) of ETN; 1:1000 (0.04 mg/mL), 1:100 (0.4 mg/mL), and 1:10 (4 mg/mL) of ADA; and 1:1000 (0.1 mg/mL), 1/100 (1 mg/mL), and 1/10 (10 mg/mL) of IFX. IDTs were read at 20 min and 24, 48, and 72 h and considered positive if the size of the initial wheal had increased by at least 3 mm in diameter and was surrounded by erythema. Immediate readings of the IDT were performed at 20 min. Skin tests were conducted at the Department of Immunology and Allergic Diseases.

### 2.3. Acute phase reactants

ESR of 0–20 mm/h and CRP of 0–5 mg/L were considered normal levels.

### 2.4. Statistical analysis

Statistical analyses were performed using SPSS Statistics Version 15 (SPSS Inc, Chicago, IL, USA). Variables were investigated using visual (histograms and probability plots) and analytical methods (Kolmogorov–Smirnov and Shapiro–Wilk tests) to determine whether the data were normally distributed. Numeric values were expressed as mean (SD), whereas nominal values were given as a number (percentage). An overall p < 0.05 was considered to have statistical significance.

## 3. Results

The 69 patients with AS were predominantly male. Systemic HSRs developed in 14 patients and ISRs in 14 patients. The 46 patients with RA were predominantly female. Systemic HSRs developed in 7 patients and ISRs in 12 patients. The other demographic data are included in Table 1.

HSRs to anti-TNFs developed in 47 of the 115 patients (40.9%) (18.3% immediate-type HSRs, 22.6% ISRs). Skin testing, both the SPT and IDT, was performed in the entire group, in other words, in all 115 patients (whether they developed HSR or not). IDTs were positive in 31 of the 47 patients who developed HSRs to anti-TNFs.

Of the 47 patients with HSRs, 21 were immediate type (44.7%) and 26 were ISRs (55.3%). IDTs were positive in two of the 21 patients (9.5%) with immediate-type HSRs and 21 of the 26 patients with ISRs (80.8%). IDT positivity was seen in the early reading; no positivity was detected in the late reading in any patient.

Of the 41 patients taking ADA, 6 developed immediate-type reactions (14.6%) and 12 developed ISRs (29.2%). IDTs were positive in one of the six patients (16.7%) with immediate-type HSRs and 10 of the 12 patients with ISRs (8.3%). SPT positivity was not detected in any of them.

Of the 40 patients taking ETN, five developed immediate-type reactions (12.5%) and 14 developed ISRs (35%). IDTs were positive in one of the five patients (25%) with immediate-type HSRs and 11 of the 14 patients with ISRs (78.6%). SPT positivity was not detected in any of them.

In 10 of the 34 (3%) patients taking IFX, immediate-type reactions developed, and IDTs and SPTs positivity were not detected in any of them, as shown in Table 2.

Twenty of the 46 patients diagnosed with RA took ADA. HSRs developed in nine of them; four of them were immediate type (only one in IDT positive early reading, no SPT positivity) and five of them were ISRs (4 IDT positive in early reading; no SPT positivity).

Seventeen of the 46 patients diagnosed with RA took ETN. HSRs developed in nine of them; two of them were immediate type (no IDT or SPT positivity) and seven of them were ISRs (five of them were IDT positive in early reading; no SPT positivity).

Nine of the 46 patients diagnosed with RA used IFX. HSRs did not develop in six of these patients. Immediate-type HSRs developed in three patients (no IDT or SPT positivity).

Twenty-one of the 69 patients diagnosed with AS took ADA. HSRs developed in nine of them; two of them were immediate type (no IDT or SPT positivity) and seven of them were ISRs (six of them were IDT positive in early reading; no SPT positivity).

Twenty-three of the 69 patients diagnosed with AS took ETN. HSRs developed in 10 of them; three of them were immediate type (only one IDT positive in early reading; no SPT positivity) and seven of them were ISRs (six of them were IDT positive in early reading; no SPT positivity).

Twenty-five of the 69 patients diagnosed with AS took IFX. HSRs did not develop in 18 of these patients. Immediate-type HSR developed in seven of them (no IDT or SPT positivity).

A statistically significant association was found between HSRs and skin test positivity in patients taking anti-TNFs (p = 0.001), as shown in Table 3. However, this relationship was not seen in patients taking IFX. The subgroup analysis showed a statistically significant association between ISRs and IDTs in immediate readings among patients taking ADA and ETN (respectively p = 0.012, p = 0.013), as shown in Table 4.

The relationship between low drug levels in the blood and ADAb positivity was statistically significant (p = 0.001). A similar association was also seen in patients taking ADA (p = 0.002). This relationship was not found in patients taking IFX or ETN, as shown in Table 5.

A statistically significant relationship was found between ADAb positivity and disease duration (p = 0.009). However, no statistically significant relationship was found between ADAb positivity and duration of anti-TNF use, DAS28, BASDAI, ESR, or CRP. When IFX, ADA, and ETN were examined separately, no relationship was found between ADAb positivity and disease duration, duration of anti-TNF use, or parameters of disease activation.

No statistically significant relationship was found between IDT positivity and serum ADAb levels. In the subgroup analysis, this relationship was not detected for ADA. Statistical calculations could not be performed for IFX or ETN.

## 4. Discussion

In the present study, the ratio of HSRs, either immediate types or ISRs, was 40.9% with a statistically significant relationship between ISRs and IDTs.

Skin tests are used to determine whether HSRs, especially ISRs, are irritant or immunological. Moreover, skin tests distinguish between IgE and non-IgE-mediated mechanisms and validate the need for a desensitization procedure for continued drug administration.

ISRs have been found in up to 37% of ETA treated patients and reported to be present in between 3.2% and 20% of ADA treated patients [10]. In our study, ISRs were found in 35% of ETN treated patients and 29.2% of ADA treated patients. A statistically significant relationship was found between ISRs and IDTs in our study. Therefore, it can be concluded that these reactions are mediated by IgE (nonirritant).

In another study, the presence of eosinophil-dominant papillary dermal edema was shown in biopsy samples from the ETN injection site, and it was concluded that ISRs may be associated with type 1 hypersensitivity [11].

In a similar study, ISRs were reported in two patients with spondyloarthropathy receiving ADA therapy. In the SPT performed in both patients, swelling and redness were detected within 15 min, suggesting the presence of type 1 hypersensitivity [12].

The important point is that, in the presence of local or systemic HSRs, desensitization can be performed if it is necessary to continue the use of medications. Case reports and case series have been reported showing that rapid drug desensitization via the subcutaneous route is effective and safe [13–16].

It is now known that antibodies may develop against TNF inhibitors and these antibodies reduce its effectiveness [17]. There are many studies on this subject [18–20]. These studies found that the antibody incidences were at different rates in different inflammatory diseases and regarding the TNF-α inhibitors used. In those studies, the rate of ADAb development against IFX varied between 6% and 83%. This rate ranged between 0.04% and 54% for ADA and between 0% and 13% for ETN [21]. These measured rates were mainly detected by IgG4 ADAbs [22]. Further, in our study, IgG subclasses were used to detect ADAbs, and the rates were 14.7% for IFX, 31.7% for ADA, and 0% for ETN, similar to those in previous studies [23,24].

After recent studies of ADAb-drug level efficacy, attention has been directed to investigating the relationship between antibody formation and developing HSRs to biological drugs. However, studies on this subject are very limited and only focus on immediate IgE-dependent reactions [25]. Some studies have determined that there may be a relationship between immediate-type reactions and ADAbs in the IgE isotype [26]. In a study in 2012, it was found that IgE-mediated reactions developed in the presence of IFX ADAb positivity, and these were ADAbs in the IgE subtype [27]. These studies support the view that there is a relationship between the development of HSRs and the presence of IgE ADAbs.

In our study, considering the relationship between ADAbs in the IgG isotypes and HSRs and the available data, the absence of an association is natural. In addition, analysis in 2015 examined the connection between the presence of IgG4 ADAbs and HSRs and disease activity in RA patients taking ETN, ADA, and IFX. Similar to our study, no significant relationship was found between the presence of IgG4 ADAbs and disease activity and HSRs [28].

The absence of a relationship between ADAb positivity and HSRs in our study may have been due to various reasons. One reason is that the IgE isotype was not measured; the fact that non-ADAb-mediated mechanisms are responsible may be another reason.

Non-ADAb-mediated mechanisms include those that cause an immediate-type reaction, cytokine release reaction (CRR), and complement-mediated pseudoallergic reaction (CARPA) [29].

The cytokine release reaction (CRR) is an immediate HSR, and is thought to be an ADAb-free and antibody-independent reaction that results in intense cytokine release. CRRs are often triggered by infections, chemotherapy drugs, monoclonal antibodies, antibiotics, and stem cell transplantation. TNF-α and interferon gamma are released with the activation of T cells, and proinflammatory cytokines appear with the activation of macrophages, dendritic cells, and endothelial cells, resulting in a systemic inflammatory response. The picture emerges in a broad spectrum from hypersensitivity-like reactions to sepsis. As a result, clinical findings related to CRRs (rash, hypotension, nausea, tachycardia, etc.) are similar to those of other ADAb-dependent reactions [30].

In a CARPA, anaphylatoxins such as C3a and C5a are released with the direct activation of complements and anaphylaxis-like symptoms occur. It differs from IgE-mediated ones because it occurs upon the first exposure [31].

The most crucial point distinguishing our study from others is that it evaluated the relationship between HSRs and skin test positivity due to ETN and ADA. We found a statistically significant association between ISRs and IDTs in ADA and ETN patients. Therefore, it can be predicted that ISRs may develop in patients with a positive IDT result.

As a result, HSRs due to the use of biological agents seem to be an essential factor that limits the use of drugs in daily practice, even leading to discontinuation of the drug sometimes. The most commonly researched drug in this area is IFX and its associated acute infusion reactions. The most frequently discussed mechanism on this subject is the development of IgE-type ADAbs.

### Limitations of the study

In cases of delayed-type reaction, not performing a patch test with 10%–20% drug concentrations can be considered a limitation.

Lack of clear standardization of skin tests against biological agents may result in false positivity and negativity.

Statistical calculations were not performed for some parameters, possibly due to the inadequate number of patients.

Lack of power may result in statistical calculations not being performed for IFX and ETN.

## 5. Conclusion

In daily practice, hypersensitivity reactions to biological agents limit their use, and sometimes it may even lead to termination of treatment. This study demonstrates that patients who exhibit injection site reactions to anti-TNF drugs have notably positive results in skin testing without maintaining a direct relationship to serum levels of ADAbs. These findings may be related to various factors. One reason is that the IgE isotype was not measured; the fact that non-ADAb-mediated mechanisms are responsible may be another reason.

Our analysis did not show an association between skin test positivity (IDT) and ADAbs, suggesting that skin findings are not linked to low drug levels.

We hope that future developments in the pharmaceutical industry and further knowledge of the workings of the immune system will enlighten us and answer our remaining questions on this subject.

## Figures and Tables

**Table 1:** Demographic data of patients with rheumatoid arthritis and ankylosing spondylitis.

Variables	Rheumatoid arthritis (n = 46)	Ankylosing spondylitis (n = 69)

Sex (Female/Male (**Number (%))**	34/12 (73.9%/26.1%)	25/44 (36.2%/63.8%)

Age (years) **(min-med-max)**	52.37 ± 12.53 (24-51.50-79)	44.41 ± 9.18 (21-44-70)

Disease duration (years)	13.48 ± 9.91 (2-10-37)	8.59 ± 7.44 (0.5-6-31)

Duration of anti-TNF use (years)	2.81 ± 2.31 (0.08-2-11)	3.04 ± 2.36 (0.33-2.50-10)

ESR (mm/h)	21.58 ± 14.61 (3-17-107)	19.33 ± 13.12 (4-19.50-45)

CRP (mgr/L)	7.73 ± 7.92 (1-3-39)	6.43 ± 7.0 (1-3-39)

BASDAI	-	2.86 ± 2.09 (0.40-2.40-9)

DAS28	3.07 ± 1.32 (1-3.02-7.10)	-

**Blood drug Level (number (%)** * **)**		
Normal	34 (73.9%)	49 (71%)
Low	12 (26.1%)	20 (29%)

**Blood ADAb Level (number (%))** ^		
Negative	39 (84.8%)	58 (84.1%)
Positive	7 (15.2%)	11 (15.9%)

**Number of patients taking anti-TNF (%)**		
Infliximab (IFX)	9 (19.6%)	25 (36.2 %)
Adalimumab (ADA)	20 (43.5%)	21 (30.4%)
Etanercept (ETN)	17 (37 %)	23 (33.3%)

**HSRs anti-TNFs**		
No	25 (54.3%)	43 (62.3%)
Yes	21 (45.6%)	26 (37.6%)

**Prick test positivity**		
No	46 (100%)	68 (98.6%)
Yes	0 (0%)	1 (1.4%)

**Intradermal test positivity (IDT)**		
No	35 (76%)	49 (71%)
Yes	11 (23.9%)	20 (29%)

Min-med-max: minimum-median-maximum. Anti-TNF: antitumor necrosis factor. ESR: erythrocyte sedimentation rate. CRP: C-reactive protein. BASDAI: Bath Ankylosing Spondylitis Disease Activity Index. DAS28: Disease activity score. ADAb: antidrug antibody. HSRs: hypersensitivity reactions.

* The drug levels for IFX, ADA, and ETN were defined as follows: ≤0.035 μg /mL (low) and ≥0.035 μg /mL (normal).

^ ADAb levels were defined as follows: for IFX, ≤2 AU/mL (negative) and >2 AU/mL (positive); for ADA, ≤3.5 AU/mL (negative) and >3.5 AU/mL (positive); and for ETN, ≤142 AU/mL (negative) and >142 AU/mL (positive).

**Table 2:** Clinical presentations of hypersensitivity reactions, and skin test positivity according to the anti-TNFs.

Patients	Immediate HSRs	ISRs	Total

**Total: 115**	21 (44.7%)	26 (55.3%)	47 (40.9%)
**SPT positivity**	0 (0%)	0 (0%)	0 (0%)
**IDT positivity**	2 (9.5%)	21 (80%)	23 (49%)

**Adalimumab (n = 41)**	6 (14.6%)	12 (29.2%)	18 (44%)
**SPT positivity**	0 (0%)	0 (0%)	0 (0%)
**IDT positivity**	1 (16.7%)	10 (8.3%)	11 (61%)

**Etanercept (n = 40)**	5 (12.5%)	14 (35%)	19 (47.5%)
**SPT positivity**	0 (0%)	0 (0%)	0 (0%)
**IDT positivity**	1 (25%)	11 (78,6%)	12 (63.1%)

**Infliximab (n = 34)**	10 (12.5%)	-	10 (12.5%)
**SPT positivity**	0 (0%)	-	0 (0%)
**IDT positivity**	0 (0%)	-	0 (0%)

ISRs: injection-site reactions. SPT: skin prick test. IDT: intradermal test.

**Table 3:** Relationship of hypersensitivity reactions (HSRs) and intradermal tests (IDTs) positivity.

	IDT negative	IDT positive	p

	Number (%)	Number (%)	

**Infliximab**			
No history of HSRs	24 (70.6%)	0	*
History of HSRs	10 (29.4%)	0	*
Total	34 (100%)	-	-

**Adalimumab**			
No history of HSRs	21 (65.6%)	2 (22.2%)	**0.020**
History of HSRs	11 (34.4%)	7 (77.8%)	
Total	32 (100%)	9 (100%)	

**Etanercept**			
No history of HSRs	15 (83.8%)	6 (27.3%)	**0.001**
History of HSRs	3 (16.7%)	16 (72.7%)	
Total	18 (100%)	22 (100%)	

**All anti-TNF takers**			
No history of HSRs	60 (71.4%)	8 (25.8%)	**0.001**
History of HSRs	24 (28.6%)	23 (74.2%)	
Total	84 (100%)	31 (100%)	

* Statistical tests could not be performed.

**Table 4:** Relationship between immediate-type reactions and injection-site reaction (ISR) and IDT (intradermal test) positivity.

Adalimumab	IDT negative Number (%)	IDT positive Number (%)	p
History of HSRs	11 (61.1%)	7 (38.9)	**0.012**
ISRs	2 (16.7%)	10 (83.3%)	
Immediate-type reactions	5 (83.3%)	1 (16.7%)	
**Etanercept**			**0.013**
History of HSRs	3 (15.8%)	16 (84.2%)	
ISRs	3 (21.4%)	11 (78.6%)	
Immediate-type reactions	4 (80%)	1 (20%)	

**Table 5:** Blood ADAb level and drug level relationship.

	Blood ADAb level negative (n = 97) Number (%)	Blood ADAb level positive (n = 18) Number (%)	p

**Blood drug level** *			**0.001**
Normal	78 (80.4%)	5 (27.8%)	
Low	19 (19.6%)	13 (72.8%)	
Total	97 (100%)	18 (100%)	

**Blood drug level of patients taking adalimumab (ADA)**			
Normal	24 (85.7%)	5 (38.5%)	
Low	4 (14.3%)	8 (61.5%)	
Total	28 (100%)	13 (100%)	**0.002**

ADAb: antidrug antibody.

* The drug levels for IFX, ADA, and ETN were defined as follows: ≤0.035 μg/mL (low) and ≥0.035 μg/mL (normal).

& Statistical tests could not be performed in patients taking IFX and ETN.
